# Variations of plasma oxidative stress levels in male patients with chronic schizophrenia. Correlations with psychopathology and matrix metalloproteinase-9: a case-control study

**DOI:** 10.1186/s12888-023-05479-0

**Published:** 2024-01-03

**Authors:** Haidong Yang, Caiyi Zhang, Man Yang, Junjun Liu, Yuting Zhang, Dongliang Liu, Xiaobin Zhang

**Affiliations:** 1 Medical College of Soochow University, 215137 Suzhou, PR China; 2 Department of Psychiatry, The Fourth People’s Hospital of Lianyungang, The Affiliated KangDa College of Nanjing Medical University, 222003 Lianyungang, P.R. China; 3 Suzhou Psychiatric Hospital, Institute of Mental Health, The Affiliated Guangji Hospital of Soochow University, 215137 Suzhou, P.R. China; 4 Department of Psychiatry, The Affiliated Xuzhou Oriental Hospital of Xuzhou Medical University, 221004 Xuzhou, China

**Keywords:** Schizophrenia, Redox dysregulation, Antioxidant, Neuroinflammation, Matrix metalloproteinase-9, Oxidative stress, Antioxidant enzymes

## Abstract

**Background:**

Accumulating evidence has indicated that oxidative stress (OS) and matrix metalloproteinase-9 (MMP-9) may contribute to the mechanism of schizophrenia. In the present study, we aimed to evaluate the associations of OS parameters and MMP-9 levels with psychopathological symptoms in male chronic schizophrenia patients.

**Methods:**

This study was an observational, cross-sectional, retrospective case-control study. Plasma hydrogen peroxide (H_2_O_2_), malondialdehyde (MDA), superoxide dismutase (SOD), catalase (CAT), glutathione peroxidase (GSH-Px), serum matrix metalloproteinase-9 (MMP-9), and tissue inhibitors of metalloproteinases-1 (TIMP-1) levels were assayed in 80 male patients with chronic schizophrenia and 80 matched healthy controls. Schizophrenia symptoms were assessed by the Positive and Negative Syndrome Scale (PANSS). Multivariate regression was used to analyze relationships between OS parameters and MMP-9, and clinical symptoms.

**Results:**

Our results demonstrated that levels of antioxidant enzymes, SOD, GSH-Px, H_2_O_2_, and MDA were significantly decreased, whereas CAT and MMP-9 levels were increased in patients with schizophrenia, when compared with healthy controls (all *P* < 0.05). In schizophrenia patients, correlation analyses showed that H_2_O_2_ levels were significantly and positively correlated with PANSS positive scores, CAT and MDA levels were significant negatively correlated with PANSS negative scores and PANSS total scores, and MDA levels were significantly positively correlated with MMP-9 levels (all *P* < 0.05). However, we did not find that MMP-9 played an interaction role between OS parameters and PANSS total scores and subscales scores (all *P* > 0.05).

**Conclusions:**

Our results showed that alterations of plasma OS parameters in male patients with chronic schizophrenia were associated with psychopathology and MMP-9, suggesting that OS and neuroinflammation may play important role in the mechanism of schizophrenia.

## Introduction

Schizophrenia is a severe mental disorder characterized by high disability, significant heterogeneity, insidious onset, prolonged duration, and a propensity for chronicity [1]. Among the various mental disorders, schizophrenia stands out as one that imposes a substantial burden of illness, second only to depression [2, 3]. Schizophrenia is a common and debilitating disorder that affects approximately 1% of the global population, with an age-standardized prevalence of 287.4 per 100,000 people [3, 4]. Previous studies have suggested that the lifetime prevalence of mental disorders in China may be estimated at 15.8-16.6%, and the lifetime prevalence and 12-month prevalence of schizophrenia were both 0.6% [5, 6]. However, these results may be underestimated, partly because of the stigma attached to the illness, and partly owing to the underdiagnosis and undertreatment of the illness in community hospitals, which results in the chronicity of the illness. However, despite extensive research efforts, the underlying pathological and physiological mechanisms responsible for schizophrenia remain largely unknown. This gap poses a hindrance to the development of effective interventions, perpetuating the chronicity of patients and exacerbating the burden of the disease.

Oxidative stress (OS) is a physiological imbalance between the production of reactive oxygen species (ROS) and the capacity of the antioxidant defense system to neutralize them [7]. ROS, which encompass free radicals and other reactive molecules, while the primary one is hydrogen peroxide (H_2_O_2_), are inherent byproducts of cellular metabolism and serve vital functions in diverse physiological processes [8]. Catalase (CAT) and glutathione peroxidase (GSH-Px) regulate its concentration by breaking down H_2_O_2_ to prevent toxic reactions [9]. Nevertheless, an abnormal buildup of ROS can result in oxidative damage to cellular components, encompassing lipids, proteins, and DNA, resulting in cell necrosis and apoptosis [10]. Numerous earlier studies including clinical and nonclinical have demonstrated the contribution of OS imbalance, such as CAT, superoxide dismutase (SOD), GSH-Px, and malondialdehyde (MDA), to the pathophysiological mechanisms of schizophrenia [11–13].

Furthermore, studies have reported significantly elevated levels of lipid peroxidation (LPO), inducible nitric oxide synthase (iNOS), and tumor necrosis factor-α (TNF-α) in restraint stress rat models, suggesting a relationship between neuropsychiatric disorders and OS and neuroinflammation [14]. NOS knock-out mice show schizophrenia-related behavioral deficits, and the atypical antipsychotic drug, Olanzapine, can alter NOS levels, accompanied by increased social interactions and reversal of behavioral deficits [15]. Moreover, interactions between inflammatory cytokines such as tumor necrosis factor-α (TNF-α) and MDA are risk factors in first episode drug-naïve schizophrenia, suggesting that inflammatory factors and OS and their interactions are involved in the pathogenesis of schizophrenia [16]. Several studies have suggested that the relationship between OS and neuroinflammation is inextricably linked to matrix metalloproteinase (MMP)-9. ROS activates MMP-9, and variations in MMP-9 levels play key roles between OS and neuroinflammatory processes [17, 18]. A case control study also reported that serum MMP-9 was correlated with MDA and total antioxidant status in drug-free male schizophrenia [19].

MMPs belong to a family of zinc-dependent endopeptidases consisting of 28 members whose abnormal function may contribute to autoimmune diseases, cancer, cardiovascular diseases, neuroinflammatory, and neurodegenerative disorders [20], among which MMP-9 is known to regulate neurotransmitter plasticity, synaptic plasticity, perineuronal net formation and integrity, myelination, neuroimmune and inflammatory cell migration [18]. Moreover, MMP-9 is considered to be an important neuroinflammatory factor that can be regulated by endogenous tissue inhibitors of metalloproteinases (TIMP)-1, and an imbalanced MMP-9 or TIMP-1 or MMP-9/TIMP-1 ratio is associated with a variety of diseases such as inflammation-accompanied cholesteatoma, chronic venous disease, atherosclerosis, and colo-rectal disease [21, 22]. Schoretsanitis et al. reviewed evidence that MMP-9 blood levels were significantly upregulated and related to disease severity in schizophrenia spectrum disorders [23]. Rahimi et al. found that the ratio of MMP-9/TIMP-1 was significantly different in a schizophrenia patient group versus the healthy control group [24].

Although increasing evidence suggests that OS and MMP-9 expression levels are altered in patients with schizophrenia, the results are inconsistent. For example, studies found no significant differences in oxidant and antioxidant enzymes including CAT, GSH, GSH-Px, SOD, NO, LPO, and total antioxidant status in schizophrenia [25, 26]. A previous study reported that serum MMP-9 levels neither differed significantly between patients with schizophrenia and healthy controls nor correlated with disease severity [27]. OS and immune inflammation and their interactions in the pathophysiological mechanisms of schizophrenia have therefore been the topics of intense investigations.

As previously mentioned, we hypothesized that OS parameters including H_2_O_2_, MDA, Mn-SOD, CuZn-SOD, total-SOD (T-SOD), CAT, GSH-Px, MMP-9, and TIMP-1 may be involved in the underlying pathophysiology of schizophrenia. Hence, we recruited male patients with chronic schizophrenia to determine (1) whether plasma OS parameters and serum MMP-9 and TIMP-1 differed between schizophrenia patients and healthy controls; (2) whether alterations in plasma OS parameters and serum MMP-9 and TIMP-1 were related to the severity of clinical symptoms; (3) whether serum MMP-9 or TIMP-1 and plasma OS parameters levels were independently or interactively correlated with clinical features. To our knowledge, this is the first study to report the relationships between OS status and MMP-9, TIMP-1, and clinical symptoms in male patients with schizophrenia.

## Subjects and methods

### Subjects and assessments

This study was an observational, cross-sectional, retrospective study using a case-control design, conducted from October 2018 to October 2020. Participants for our study were recruited via community advertisements and referrals from local hospitals. Each participant was thoroughly briefed about the purpose and procedures of the study and signed an informed consent form, agreeing to their data being used for research purposes. Data on general information, sociodemographic characteristics and medical history were collected using face-to-face interviews with guardians of the participants or the participants themselves. A total of 80 male inpatients with schizophrenia who had been hospitalized continuously for more than 2 years and taking a stable dose of oral antipsychotic medication for at least 12 months prior to the study, were recruited from Lian Yun Gang Fourth People’s Hospital and its Medical Group Mental Disorder Department. This study included only male subjects due to the higher prevalence of long-term hospitalization, earlier onset, and more severe symptoms among male schizophrenia patients in China [28, 29]. Additionally, recent findings indicate higher SOD activity in male patients with earlier stage schizophrenia [30]. Furthermore, hormonal influences on OS could potentially confound our results [31]. Patients with schizophrenia were confirmed by using the Structured Clinical Interview of the Diagnostic and Statistical Manual-IV. A questionnaire was used to collect general patient data such as age, education, smoking, body mass index (BMI), age of onset, and duration of illness. The antipsychotic medication doses were converted to the equivalent doses of chlorpromazine. The severity of psychotic symptoms was evaluated by two experienced psychiatrists using the positive and negative syndrome scale (PANSS). The inter-rater correlation coefficient for the PANSS score was > 0.8.

The patients’ inclusion criteria were: (1) males, aged 18–60 years, of Han ethnicity; (2) hospitalized for more than 2 years; (3) stable on antipsychotic medication for 12 months;(4) had not taken or were not receiving anti-inflammatory or antibiotic treatment 4 weeks prior to inclusion; and (5) all participants had at least completed primary school education and were able to understand the questions asked by the investigators.

Exclusion criteria for all enrolled patients were comorbid neurological disorders such as mental retardation, dementia, epilepsy, degenerative disease, traumatic brain injury, and endocrine disorders such as thyroid dysfunction, diabetes mellitus, alcoholic, substance dependence, or abuse. Additionally, participants who had not completed primary school education were also excluded.

Eighty healthy controls matched for age, sex, education, BMI, and smoking were recruited by advertisements from the community in Lian Yun Gang. Healthy controls did not meet the diagnoses of Diagnostic and Statistical Manual-IV (DSM-IV) Axis I criteria for a major disease, and exclusion criteria also included a family history of mental disorder or alcohol abuse/dependence. To determine the health status of all participants, we conducted routine physical examinations and laboratory tests. These tests included the measurement of blood pressure, a complete blood count, liver and kidney function tests, blood glucose levels, triglyceride levels, and thyroid function tests. We ensured that participants who had normal results in these tests were included in our study.

Ethical considerations were paramount in our research. All participants or the guardians of the participants were fully informed about the research aims and procedures, and participation was on a voluntary basis. We ensured that all data were anonymized to protect participants’ privacy. The present protocol was approved by the Ethics Committee of Lian Yun Gang Fourth People’s Hospital, and all participants or their guardians gave informed written consent.

### Blood sampling and biochemical assays

Peripheral venous blood samples from healthy controls and patients were collected in the morning between 07:00 and 09:00 after overnight fasting. Blood samples were collected in procoagulant tubes and anticoagulant tubes, centrifuged at 3000 rpm for 15 min, serum was extracted from the procoagulant tube, and plasma was extracted from the anticoagulant tubes, then stored at -80 °C before analyses. All blood samples were assayed in duplicate by a technician who was blinded to the sample identity and clinical status. The intra- and inter-assay coefficients of variation for OS parameters ranged from 3.4 to 7.2%, and for MMP-9 and TIMP-1, they ranged from 2.2 to 6.3%.

The plasma levels of H_2_O_2_, MDA, Mn-SOD, CuZn-SOD, T-SOD, CAT, and GSH-Px were measured in accordance with a commercially available kit (Nanjing Jiancheng Bioengineering Institute, Nanjing, China). The levels of H_2_O_2_ (mmol/L) and GSH-Px (U/mL) were assessed using a colorimetric method, MDA (nmol/mL) analysis used a thiobarbituric acid method, Mn-SOD (U/mL), CuZn-SOD (U/mL), and T-SOD (U/mL) involved hydroxylamine method, and CAT (U/mL) was determined using a visible light method [32, 33]. Serum MMP-9 (ng/mL) and TIMP-1 (ng/mL) levels were tested using Luminex liquid suspension chip detection according to the manufacturer’s instructions (R&D Systems, Minneapolis, MN, USA).

### Statistical analysis

All statistical data were performed using the SPSS statistical software for Windows, version 19.0 (SPSS, Chicago, IL, USA). The sample sizes were calculated using Gpower 3 v3.1.9.7 (http://www.ats.ucla.edu/stat/gpower/). A “means: Difference between two independent means (two groups)” test was used, with a desired power of 90%, at a 10% significance level, and an effect size of 0.8 [34]. The normal distribution of variables was examined using the Kolmogorov-Smirnov test. Continuous variables and normality distribution were calculated using Student’s *t*-test, and were expressed as the mean ± standard deviation (SD). Continuous variables and non-normally distributed data were expressed as the median (25th quartile and 75th quartile). The chi-square test was used for categorical variables. We transformed the non-normally distributed serum MMP-9, TIMP-1, and MDA levels into normally distributed data by taking the natural logarithm. A power analysis was performed as part of the study design to ensure a sufficient sample size to detect a large effect size. Cohen’s d values were used to report the effect size, where 0.2 was considered a small effect size, 0.5 was a medium effect size, and 0.8 was a large effect size. The results were considered statistically significant when *P* < 0.05.

Multiple analysis of covariance (MANCOVA) was first used to test the differences in levels of H_2_O_2_, MDA, SOD, CAT, GSH-Px, MMP-9, and TIMP-1 between the patient group and healthy controls. In this model, OS parameters and MMP-9 and TIMP-1 levels were dependent variables, and the diagnoses (patients and healthy controls) were set as fixed factor and age, with education, BMI, and smoking as covariates. Each OS parameter and MMP-9 and TIMP-1 levels were set as dependent variables, while the independent variables were the diagnosis, covariates were age, education, BMI, and smoking, and analysis of covariance (ANCOVA) was used to test the difference between the patient and healthy control groups. Multiple comparisons were adjusted using the Bonferroni correction. The correlation of normally distributed data was performed using the Pearson’s correlation test, and non-normally distributed data used Spearman’s correlation. Exploratory multivariate regression was used to analyze the relationships between each dependent variable of OS parameters and MMP-9, TIMP-1, and total scores of PANSS and subscales, with demographic features as covariates.

## Results

### Comparison of demographic and general clinical data

Table 1 shows the demographic information and clinical data between the schizophrenia and healthy control groups. We found no significant relationship in age, education, BMI, and smoking between patients and controls (*P* > 0.05). The age of onset of schizophrenia patients was 26.99 ± 8.40 years, with a total PANSS score of 58.09 ± 14.90, a positive symptom score of 11.03 ± 4.55, a negative symptom score of 29.24 ± 6.37, a general psychopathology score of 17.83 ± 7.18, and a chlorpromazine equivalent dose of 589.13 ± 195.19 mg/d.

**Table 1:** Demographics of patients with schizophrenia and healthy controls (mean ± SD)

	patients (n = 80)	controls (n = 80)	*t/χ* ^*2*^	*P*
Age (years)	40.62 ± 9.80	39.81 ± 9.56	0.531^a^	0.596
Education (years)	9.10 ± 2.85	9.96 ± 3.27	-1.779^a^	0.077
BMI (kg/m^2^)	24.51 ± 3.66	25.37 ± 2.97	-1.637^a^	0.104
Smoking	39 (48.8%)	41 (51.2%)	2.525^b^	0.112
Age of onset (years)	26.99 ± 8.40	-	-	-
Duration of illness (years)	11.0 (7.0, 18.9) ^c^	-	-	-
PANSS total score	58.09 ± 14.90	-	-	-
P subscores	11.03 ± 4.55	-	-	-
N subscores	29.24 ± 6.37	-	-	-
G subscores	17.83 ± 7.18	-	-	-
Equivalent dose of chlorpromazine (mg/d)	589.13 ± 195.19	-	-	-

BMI, body mass index; PANSS, positive and negative syndrome scale

^a^Student’s *t*-test

^b^χ^2^ test

^c^Median (25th quartile and 75th quartile)

### Levels of MMP-9, TIMP-1, and OS parameters between schizophrenia patients and healthy controls

MANCOVA analyses showed that the effect of diagnosis was significant (*F* = 36.817, *P* < 0.001). ANCOVA results showed the difference in plasma OS parameters and serum MMP-9 levels between patients and healthy controls after covarying for age, education, BMI and smoking, as shown in Table 2. The Mn-SOD (*F* = 110.618, *P* < 0.001), CuZn-SOD (*F* = 7.499, *P* = 0.007), T-SOD (*F* = 53.010, *P* < 0.001), GSH-Px (*F* = 37.778, *P* < 0.001), H_2_O_2_ (*F* = 9.881, *P* = 0.002), and MDA (*F* = 130.510, *P* < 0.001) levels were significantly lower in patients than in healthy controls. The CAT and MMP-9 levels were significantly higher in patients, when compared with healthy controls (*F* = 15.613, *P* < 0.001; *F* = 6.271, *P* = 0.013; respectively). No significant difference of TIMP-1 levels was found between the two groups (*P* = 0.799). The differences remained significant after Bonferroni correction for Mn-SOD, CuZn-SOD, T-SOD, CAT, GSH-Px, H_2_O_2_, and MDA levels (*P* < 0.05). However, the levels of MMP-9 were not significantly different between patients and healthy controls after multiple corrections (*P* > 0.05).

**Table 2:** The levels of OS parameters and MMP-9 and TIMP-1 between patients and healthy controls

	patients	controls	*F*	*P*
Mn-SOD (U/ml)	4.49 ± 1.94	8.62 ± 3.04	110.618	0.000
CuZn-SOD (U/ml)	11.55 ± 3.28	12.99 ± 3.36	7.499	0.007
T-SOD (U/ml)	16.04 ± 4.47	21.61 ± 5.20	53.010	0.000
CAT (U/ml)	3.21 ± 0.59	2.75 ± 0.93	15.613	0.000
GSH-Px (U/ml)	68.86 ± 13.04	86.25 ± 20.45	37.778	0.000
H_2_O_2_ (mmol/L)	64.16 ± 24.28	79.99 ± 31.69	9.881	0.002
MDA^◊^ (nmol/L)	0.76 ± 0.19	1.05 ± 0.11	130.510	0.000
MMP-9^◊^(ng/mL)	1.37 ± 0.34	1.24 ± 0.32	6.271	0.013
TIMP-1^◊^ (ng/mL)	1.50 ± 0.12	1.49 ± 0.11	0.065	0.799

SOD, superoxide dismutase; CAT, catalase; GSH-Px, glutathione peroxidase; H_2_O_2_, hydrogen peroxide; MDA, malondialdehyde; MMP-9, matrix metalloproteinase; TIMP, tissue inhibitors of metalloproteinases. ◊, the result of natural logarithm transformations

### The relationship between OS parameters, MMP-9 and clinical symptoms in patients with schizophrenia

In patients with schizophrenia, correlation analyses showed that H_2_O_2_ concentration was significant positively associated with the PANSS positive symptoms score (*r* = 0.307, *P* = 0.006), CAT activities and MDA levels were significant negatively correlated with the PANSS negative symptoms score (*r* = -0.366, *P* = 0.001; *r* = -0.241, *P* = 0.032; respectively) and the PANSS total score (*r* = -0.302, *P* = 0.006; *r* = -0.271, *P* = 0.015; respectively). The MDA levels was significant positively associated with MMP-9 levels (*r* = 0.312, *P* = 0.005). Smoking was negatively related to H_2_O_2_ levels (*r* = -0.226, *P* = 0.043). However, there was no significant association between chlorpromazine equivalent dose and each OS parameter and MMP-9 (*P* > 0.05).

**Fig. 1:**
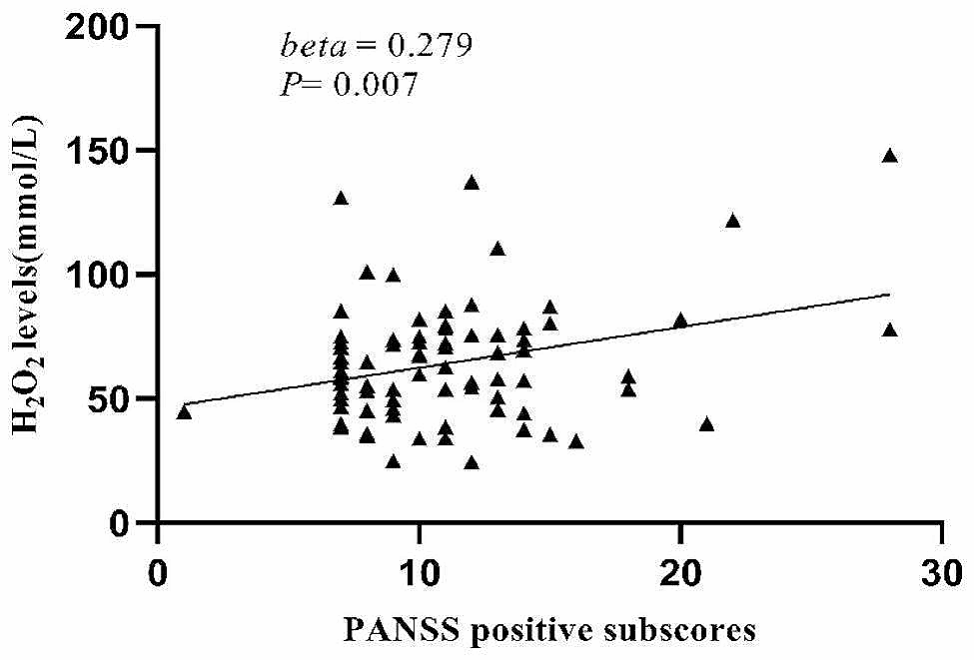
Correlations between H_2_O_2_ levels and PANSS positive subscores

**Fig. 2:**
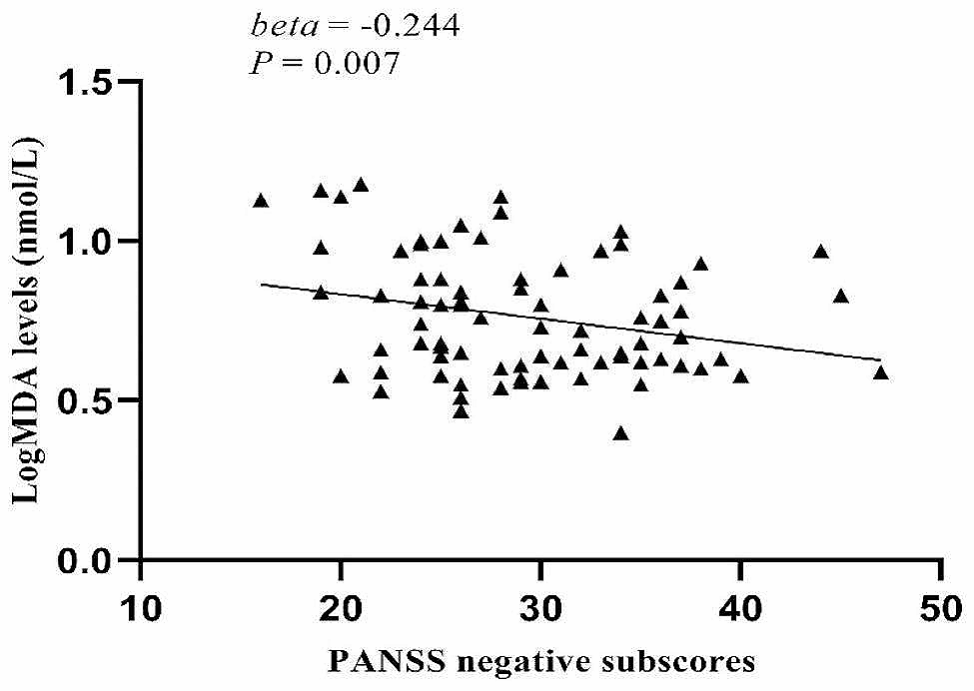
Correlations between log MDA levels and PANSS negative subscores

**Fig. 3:**
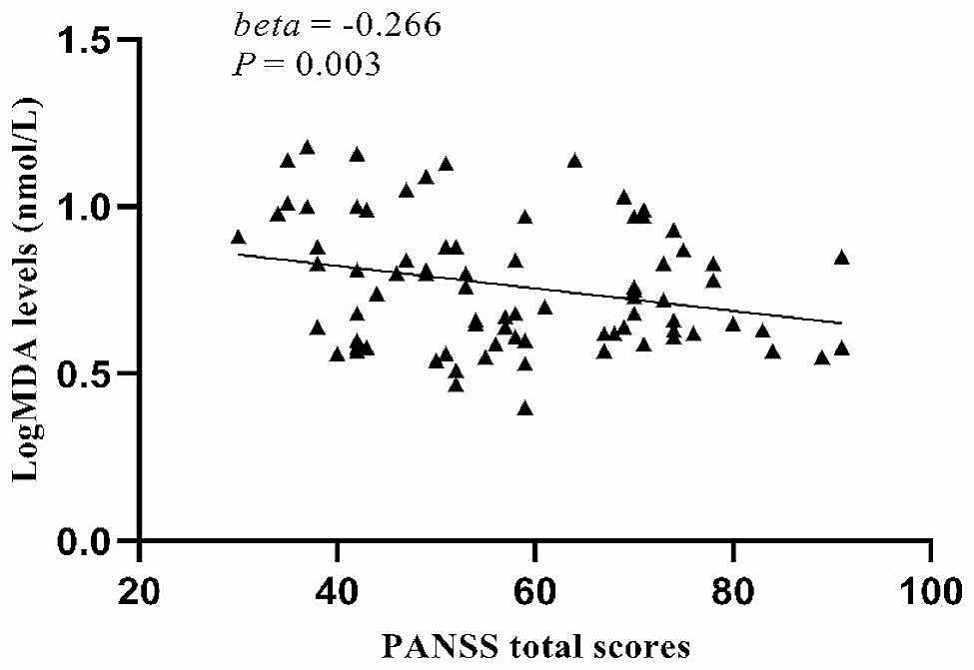
Correlation between log MDA levels and PANSS total scores

Age, education, BMI, smoking, equivalent dose of chlorpromazine, age of onset, and duration of illness were set as independent variables and potential confounding factors, and stepwise multiple regression analysis demonstrated that H_2_O_2_ levels were significantly correlated with PANSS positive symptom scores (beta = 0.279, *t* = 2.759, *P* = 0.007), CAT activities and MDA levels were significantly correlated with the PANSS negative symptoms score (beta = -0.185, *t* = -2.030, *P* = 0.046; beta = -0.244, *t* = -2.788, *P* = 0.007; respectively), and MDA levels were significantly correlated with the PANSS total score (beta = -0.266, *t* = -3.093, *P* = 0.003). We did not find that MMP-9 had interactive effects between OS parameters and PANSS total scores and subscales scores (*P* > 0.05). Using Bonferroni *post hoc* comparisons, H_2_O_2_ levels were correlated with PANSS positive subscores (Fig. 1), and MDA levels were correlated with the PANSS negative subscores (Fig. 2) and with PANSS total scores (Fig. 3) (*P* < 0.05).

## Discussion

The present study showed that activities of Mn-SOD, CuZn-SOD, T-SOD, GSH-Px, and levels of H_2_O_2_, MDA were lower, whereas CAT activity and MMP-9 levels were higher in patients with schizophrenia than in healthy controls. However, no significant difference was observed in serum TIMP-1 levels between the schizophrenia and healthy control groups. Furthermore, H_2_O_2_ levels were positively correlated with PANSS positive subscores, while MDA levels were negatively correlated with PANSS negative subscores and PANSS total scores, and MMP-9 levels were correlated with MDA levels. To date, this is the first study to report the relationship between OS parameters and MMP-9 in male patients with chronic schizophrenia of Chinese Han ethnicity.

The present study showed alterations of OS parameters levels in patients with schizophrenia, which is consistent with numerous previous studies, suggesting that oxidation and antioxidant reactions were involved in the pathophysiology of schizophrenia [35–37]. However, the reported results regarding antioxidant enzyme levels were contradictory. For example, Zhang et al. found that MDA levels were increased and no significant difference in CAT levels in patients with chronic schizophrenia compared to healthy controls was observed [38]. Other studies demonstrated that MDA, SOD, CAT, and GSH-Px levels were significantly elevated in patient groups [39, 40]. Notably, many factors may result in differences in OS parameters, such as age, diet, smoking, sample size, method of sampling for assays, (i.e., serum or plasma), different stages of disease, antipsychotic medications used, duration of illness, and sex [41, 42]. Nonetheless, these studies provided further evidence from different perspectives of the relationships between the imbalances of OS parameters and schizophrenia. In the central nervous system, proper redox homeostasis disrupts the maintenance of membrane potential, synthesis of neurotransmitters such as glutamate, dopamine (D), gamma-aminobutyric acid, N-methyl-D-aspartate (NMDA), synaptic connectivity and plasticity, and is also involved in astrocyte and mitochondrial metabolism [43, 44]. Animal models of schizophrenia indirectly demonstrated the relationship between increased activities of SOD and CAT and behavioral deficits in rats [45]. Our study found abnormal plasma Mn-SOD, CuZn-SOD, T-SOD, GSH-Px, H_2_O_2_, and MDA levels in patients with schizophrenia, but the exact pathophysiological process remains unclear and warrants further investigation.

Previous studies have shown that OS had significant effects on clinical symptoms of schizophrenia. For example, Boskovic et al. found that the polymorphism of CATc.66 + 78 C > T was related with negative PANSS scores [46]. Bai et al. indicated that serum SOD activity was negatively associated with positive symptoms [36]. Hendouei et al. found that serum MDA levels were correlated with all PANSS subscales, and GSH levels were negatively associated with PANSS negative scores in patients with chronic schizophrenia [47]. Furthermore, our study found that decreased H_2_O_2_ levels were positively related to positive symptoms, and that decreased MDA levels and elevated CAT activities were negatively associated with negative and total PANSS symptoms. It is well known that H_2_O_2_ is considered a direct marker of OS, and that MDA is considered an indirect marker of OS. Disturbances in the balance between the antioxidant enzymes CAT, SOD, and GSH-Px and H_2_O_2_ and MDA lead to OS, and antioxidant therapy has been shown to be effective in improving symptoms of schizophrenia [48, 49]. Evidence has shown that doxycycline, and antioxidant and anti-inflammatory agents, attenuated ketamine-induced schizophrenia-like behavior and enhanced the therapeutic effect of the antipsychotic risperidone in a mouse model of schizophrenia [50]. Several hypotheses have been proposed; for example, metabolism of the neurotransmitters dopamine and glutamate generates high levels of ROS that affect synaptic plasticity and signaling through NMDA and D2 receptors, and that imbalance in oxidative and antioxidant systems lead to DNA methylation and drive parvalbumin interneuron damage, which is associated with the symptoms of schizophrenia [51–53]. These results demonstrated that H_2_O_2_, CAT, and MDA may play important roles in the severity of clinical symptoms, indicating that changed H_2_O_2_, CAT, and MDA levels are correlated with pathophysiology of schizophrenia.

Our finding showed a significant increase of MMP-9 levels in patients with schizophrenia, when compared with healthy controls, which is consistent with previous studies. [54, 55] There was a study showing that the C/C genotype and C allele predominant on single nucleotide polymorphisms of MMP-9 in schizophrenia [56]. Another study indicated that expression of the *MMP-9* gene was higher and negatively related to DNA methylation in deficit schizophrenia [57]. A preliminary magnetic resonance imaging study reported that smaller left and right hippocampal volumes were negatively associated with higher MMP-9 plasma levels [58]. Studies have also reported that neuroglia and neuronal cells in the brain secreted MMP-9, and aberrant MMP-9 produced deleterious effects such as neuroinflammation, neurotoxicity, and weakening of the integrity of the blood-brain barrier [59]. These results suggested that dysregulations of MMP-9 may contribute to the process of schizophrenia.

We also found that MDA levels were positively correlated with MMP-9 levels in schizophrenia patients. Injecting dopamine reuptake inhibitors into mice has suggested that dopamine metabolism can induce additional OS, and that OS increases MMP-9 expression via the receptor for advanced glycation end products shredding [60]. A previous review showed that neuroinflammation and OS and their interrelationships were causative factors in schizophrenia [18]. The review found that neuroinflammation increased the secretion of MMP-9, which exacerbated oxidative damage via free radicals, due to elevated MMP-9, and that pro-inflammatory cytokines were released and neuroinflammation increased, which further contributed to OS by free radical formation. Additionally, accumulating evidence has shown that the interactive effect of low brain-derived neurotrophic factor (BDNF) and CAT or GSH-Px levels were related to PANSS cognitive factor and PANSS depressive factor, respectively [38]. The interaction between TNF-α and MDA or interleukin-8 (IL-8) and MDA or IL-8 and SOD levels are associated with clinical symptoms of PANSS [61]. Collectively, oxidative damage and neuroinflammation may be jointly involved in the etiology of schizophrenia, but the exact pathway is still unknown and needs further study.

There were several limitations in our study. First, compared to first-episode and drug-naïve patients with schizophrenia, chronic hospitalized male patients had longer durations of clinical symptoms. Although we controlled for confounding factors by using statistical methods, there were still compounding factors such as smoking, medications, duration of illness, and other unidentified cause that could have potentially affected the results of the examinations. Second, OS parameters were extensively, and more parameters needed to be detected to investigate the interactions with cytokines. Third, cross-sectional studies could not identify the causal relationships between OS parameters and PANSS. Longitudinal studies are necessary to determine this possibility. Finally, we only collected data on male demographics, which may limit the results.

In conclusion, our preliminary findings provide evidence that imbalances in oxidative and antioxidant parameters and neuroinflammatory factors are potential contributors to the chronic phase of male schizophrenia. Variations of H_2_O_2_, CAT, and MDA levels were associated with the severity of clinical symptoms, and MMP-9 levels correlated with MDA levels. These results provide additional evidence that OS and MMP-9 may be implicated in the pathophysiology of schizophrenia. In future research, we suggest further characterization of the relationship and mechanisms of OS and MMP-9 in the pathophysiology of schizophrenia, across both genders. Larger-scale studies, including a significant number of female patients, could provide valuable insights into gender-specific responses and are vital for validating our results. Additionally, it would be interesting to see if our findings could be applied in clinical practice to improve the treatment of patients with schizophrenia.

## Data Availability

The datasets used and analyzed during the current study are available from the corresponding author on reasonable request.
